# Beyond access: usage variety, instrumental support, and later-life depression in a prospective study of Chinese older adults

**DOI:** 10.3389/fpubh.2026.1895216

**Published:** 2026-09-30

**Authors:** Ningyi Hou, Tianyuan Chen, Zhixian Yu

**Affiliations:** Nottingham University Business School China, University of Nottingham Ningbo China, Ningbo, China

**Keywords:** CLASS, depressive symptoms, digital divide, health equity, instrumental support, older adults, resource multiplication, sustainable development goals

## Abstract

**Introduction:**

The digital divide is treated as a social determinant of later-life mental health, yet most evidence is cross-sectional and cannot separate its prospective effect from contemporaneous confounding. We examined which facets prospectively predict depression in older adults, and through what pathway.

**Methods:**

We analyzed three waves (2018, 2020, 2023) of the China Longitudinal Aging Social Survey. Four 2020 divide indicators (access, proficiency, usage variety, outcomes) predicted 2023 CES-D-9 symptoms, adjusting for baseline symptoms and covariates; the 2018–2020 interval provided an earlier-window replication. Half-longitudinal (Cole-Maxwell) mediation tested four social-connection mediators, moderated by hukou, living arrangement, and gender.

**Results:**

After adjustment for baseline depression, only the usage-variety divide—a narrower range of online activity—predicted higher subsequent depressive symptoms (b = 0.069, 95% CI 0.008–0.131). Of four mediators, only instrumental support mediated this association (indirect 0.019, bootstrap 95% CI 0.010–0.033), replicating in the 2018–2020 window (indirect 0.014, bias-corrected 95% CI 0.005–0.028). The pathway was stronger among urban and co-resident adults, with baseline instrumental support far higher in urban than rural areas (difference 0.82, *p* < 0.001), consistent with resource multiplication rather than substitution.

**Discussion:**

Conditioning on baseline depression, only the usage-variety divide—not access, proficiency, or outcomes—is prospectively associated with later depressive symptoms (chiefly as a stable between-person difference); this association was statistically mediated by lower instrumental support. Because the mediated association was stronger in resource-rich subgroups, the pattern is more consistent with resource multiplication than with substitution. Translating digital inclusion into mental-health gains may require broadening use while strengthening instrumental-support infrastructure, especially in rural settings.

## 1 Introduction

Depression is among the leading contributors to disability and reduced quality of life worldwide, and its burden is concentrated in later life. Recent meta-analytic evidence indicates that the global prevalence of depressive symptoms among older adults is approximately 35.1% (1), and the burden is substantially higher in low- and middle-income countries, reaching a pooled prevalence of 38.8% compared with 17.1% in high-income settings (2). With the global population aged 60 and over projected to rise from 1 billion in 2020 to 2.1 billion by 2050 (3), identifying modifiable social determinants of late-life depression is a global public-health priority directly relevant to Sustainable Development Goal (SDG) 3 (Good Health and Wellbeing) and the United Nations Decade of Healthy Aging (2021–2030) (4, 5). This priority is acute in China, where an estimated 297 million citizens were aged 60 or older by the end of 2023, with elevated depressive-symptom prevalence and substantial rural-urban disparities (6, 7).

The rapid digitalization of everyday life has introduced a novel social determinant of late-life mental health: the digital divide, a new axis of social inequality in the digital era, and thus a concern for SDG 10 (Reduced Inequalities) as well as SDG 3, and for the 2030 Agenda pledge to leave no one behind (8). Originally conceived as a binary gap in internet access, it has been reconceptualized over the past two decades as a sequentially nested, multilevel construct spanning material access, usage skills and breadth, and outcome benefits (9). The widely adopted access-capability-outcome framework distinguishes a first-level access divide in connectivity and devices; a second-level capability divide, itself separable into operational skill and usage variety (10); and a third-level outcome divide in the consequences people derive from use. This stratification is salient for older adults, for whom barriers shift over time from simple access toward the skills required to use digital tools effectively and the outcomes that use does or does not produce. Because these levels are compound and sequential—access is a precondition for capability, and capability for the outcomes that use produces (11)—they are treated here as distinct, sequentially ordered dimensions that jointly define the divide, rather than as interchangeable reflections of a single latent trait.

A growing body of research links the digital divide to depressive symptoms among older adults, generally reporting that deeper divides are associated with poorer mental health. At the first level, internet access has been associated with fewer depressive symptoms (12, 13), and quasi-experimental evidence from China's Broadband China pilot has been interpreted as showing that access expansion reduces depression in middle-aged and older adults (14); digital inclusion has been linked to depression via non-cognitive abilities (15) and social engagement (16). At the second level, both operational skill (17, 18) and usage variety (19, 20) have been associated with depressive symptoms, and the two sub-dimensions are theoretically and empirically separable (10). Evidence on the third-level outcome divide is more limited in older populations; in the broader Chinese adult population it predicts wellbeing independently of access and skill (21).

A resource account clarifies why the divide should bear on mental health and specifies the pathway the present study tests. Digital inequality is increasingly understood not as a single gap but as a compound, sequential process in which access, skills, and the breadth of use are progressively linked to the tangible and social resources older adults are able to derive from being online (11). Consistent with this, longitudinal evidence indicates that internet use predicts fewer depressive symptoms in older adults largely by sustaining social resources: in the Health and Retirement Study, the effect was partly mediated by reduced loneliness and maintained social contact (22), and a random-intercept cross-lagged analysis of English longitudinal data found internet use and social resources to be reciprocally related within persons, most consistently for structural facets such as contact frequency and network size (23).

The social resources that internet use sustains are not monolithic: social connection is itself multidimensional. The integrative public-health framework articulated by Holt-Lunstad (24–26) distinguishes a structural component (the presence and quantity of relationships), a functional component (the instrumental, informational, or emotional resources relationships provide), and a qualitative component (the affective evaluation of relationships). The three components are correlated but separable, and aggregating them can obscure their differential associations with health (27, 28). For older adults, the internet plausibly bears on all three-sustaining contact, mobilizing practical help, and maintaining intimacy; the three components are nonetheless unlikely to be equally implicated. We therefore tested them competitively, but with a directional expectation. General social-support research links late-life depression most consistently to the emotional component, whereas received instrumental support shows weak or null associations (29); perceived or anticipated instrumental support-knowing that tangible help is available-is, by contrast, protective in later life (30–32). The digital divide may be related to precisely this perceived availability: in China, community and public instrumental services for older adults-home visits, medical accompaniment, shopping assistance, meals, and day-care-are increasingly accessed, coordinated, and made visible through digital channels, so that greater usage variety may help older adults remain linked to and aware of this instrumental-support ecosystem, whereas low-barrier emotional contact survives even narrow use. We accordingly expected the functional component-the perceived availability of community instrumental support-rather than the structural or emotional components, to mediate the prospective association between the usage-variety divide and depressive symptoms.

Two questions remain unresolved. First, the evidence is predominantly cross-sectional, estimating contemporaneous correlations that cannot establish temporal order; whether these associations persist once exposure is ordered before outcome and baseline depression is held constant (and whether any association reflects within-person change rather than stable between-person differences) remains largely untested in older populations. Second, the mediation pathway is under-specified: prior mediation work in Chinese aging cohorts has relied predominantly on social participation (16, 33, 34) or social-capital indices (33, 35) and, where it names a component of social connection, has defaulted to the structural one, rarely testing components competitively (36, 37); it therefore remains open which level of the divide, acting through which component, accounts for a prospective association. The present study addresses both by ordering exposure before outcome, separating between- and within-person variation, and testing the structural, functional, and qualitative components competitively, so that the operative level and mediating component are identified rather than assumed.

If this functional pathway involves community instrumental support, its strength should depend on whether such support is available in the first place, which varies systematically across older adults. Two classic accounts make opposing predictions (38). On a resource-substitution logic, a resource matters most where alternatives are scarce, so greater usage variety should help most the older adults with the least instrumental support; on a resource-multiplication logic, complementary resources compound, so it should most help those who already command the supporting infrastructure. We therefore prespecified three axes along which instrumental support and its mental-health returns are known to differ in Chinese cohorts—hukou (urban vs. rural), living arrangement (co-resident vs. empty-nest), and gender—and tested them as competing predictions of substitution vs. multiplication rather than as descriptive subgroup contrasts. Gender is a natural axis for this contrast: the substitution-multiplication logic was itself developed to explain gender differences in how a resource converts into mental health (38), and gender differences in later-life support and depression are well documented among older Chinese adults (39). Adjudicating between substitution and multiplication was thus built into the design, and identifies for whom, if anyone, greater usage variety is converted into a mental-health resource.

Against this background, we use three waves of a national cohort of older Chinese adults to address two questions. RQ1: Are the four indicators of the digital divide—access, smartphone proficiency (second-level), usage variety (second-level), and the outcome divide (third-level)—prospectively associated with later depressive symptoms once baseline depression and confounders are controlled? RQ2: Which component of social connection mediates any such association? By ordering exposure before outcome, establishing longitudinal measurement invariance, testing the components of social connection competitively, and replicating the mediation in a second window, the study provides a more stringent and mediation-specific account than the prevailing cross-sectional literature affords. We constructed the full multilevel divide-all four indicators across the three levels as the analytic object, so that no facet was privileged by design; yet our reading of prior work, in which the breadth and variety of use rather than mere access tracks later-life wellbeing (10, 19, 20), led us to expect *a priori* that the second-level usage-variety facet, with perceived instrumental support as the mediating component, would account for the prospective association.

## 2 Methods

### 2.1 Study design and data source

Data were drawn from the China Longitudinal Aging Social Survey (CLASS), a nationally representative survey of community-dwelling Chinese adults aged 60 years and older conducted by the National Survey Research Center at Renmin University of China using multistage probability sampling across 30 provinces. We analyzed the 2018, 2020, and 2023 waves. Digital-divide indicators and social-connection mediators measured in 2020 were used to predict depressive symptoms in 2023, with the 2020 values of both the outcome and the mediators held constant; the 2018-to-2020 interval served as an earlier replication window (the same cohort observed over an earlier interval, so participants largely overlap; this tests robustness across observation windows rather than in an independent sample). Conditioning each outcome on its own baseline value yields estimates of the association between the 2020 digital divide and subsequent change in depressive symptoms through 2023, rather than the contemporaneous correlations that characterize the largely cross-sectional prior literature.

The construction of the analytic samples is summarized in Figure 1. The three waves contributed 11,419 (2018), 11,398 (2020), and 11,670 (2023) respondents, who were linked across waves by personal identifier after removing records with missing identifiers or duplicates; the outer union of all linkable identifiers yielded a panel of 18,293 individuals. The main analytic sample for the prospective lagged models comprised the 5,914 respondents with a complete 2020 baseline and a valid 2023 CES-D-9 outcome (12,379 excluded for lacking a valid 2020 or 2023 outcome). Because the second- and third-level (capability and outcome) divides and the mediators are defined only among internet users, the higher-order divide and mediation models were estimated within the internet-user subsample: of the main analytic sample, 1,972 respondents used the internet at the 2020 baseline (3,942 non-users excluded), of whom 1,828 had complete data on the covariate block and contributed to the adjusted models. The earlier replication window linked the 2018 and 2020 waves, yielding 7,502 respondents observed across the interval.

**Figure 1:**
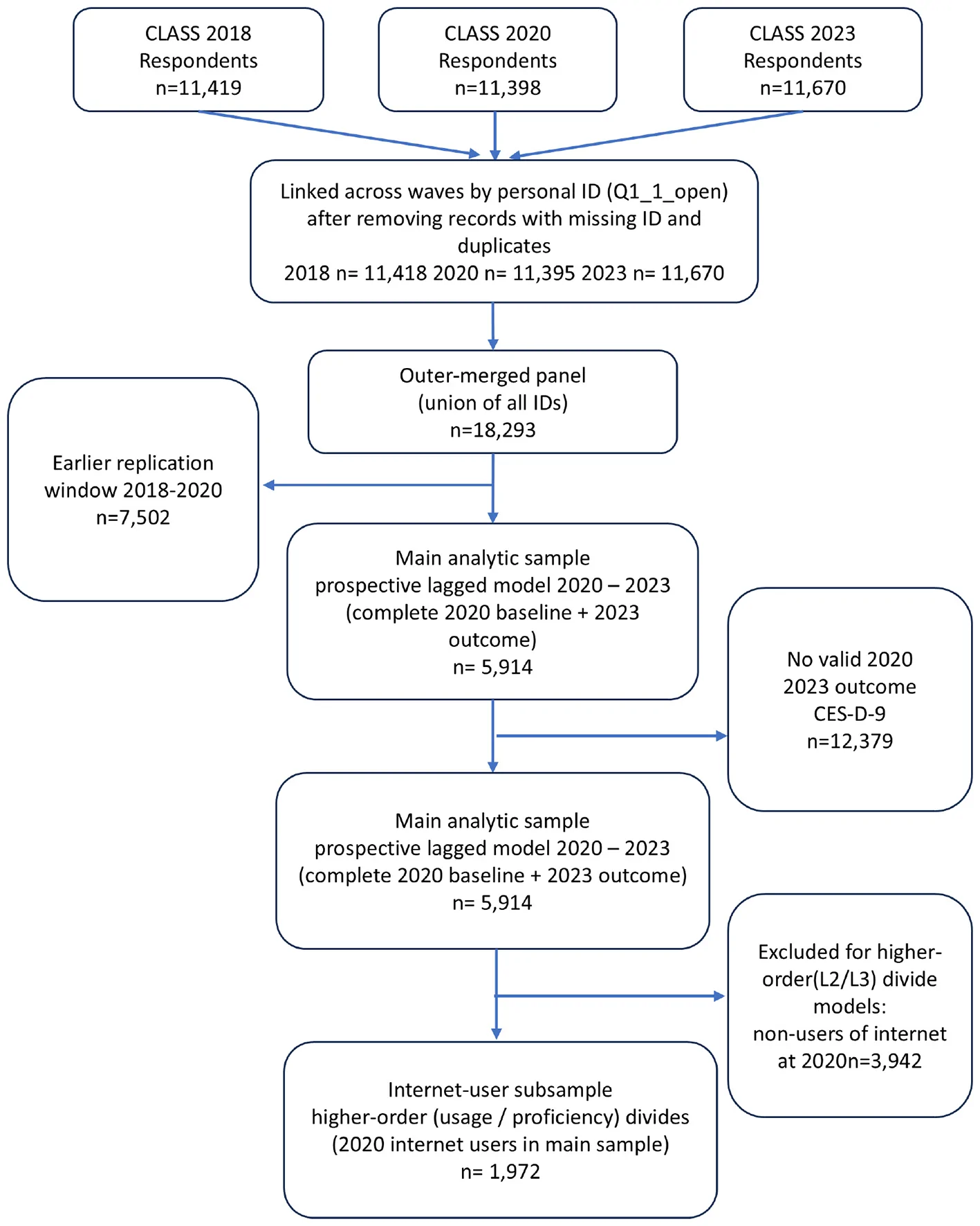
Derivation of the analytic samples. Respondents from the 2018, 2020, and 2023 waves of CLASS were linked by personal identifier into an outer-merged panel; the main analytic sample for the prospective lagged models comprised respondents with a complete 2020 baseline and a valid 2023 CES-D-9 outcome, the internet-user subsample comprised 2020 internet users within this sample (used for the capability- and outcome-level divide and mediation models), and the 2018–2020 interval defined an earlier replication window in the same cohort. CLASS, China Longitudinal Aging Social Survey; L2/L3, second-/third-level divide; CES-D-9, 9-item Center for Epidemiologic Studies Depression Scale.

### 2.2 Ethics approval

This study is a secondary analysis of publicly available, de-identified CLASS data. The original data collection received institutional ethical approval from Renmin University of China, and all participants provided written informed consent. Secondary analysis of de-identified, publicly released survey data did not require additional institutional ethics review.

### 2.3 Measures

#### 2.3.1 Outcome: depressive symptoms

Depressive symptoms were measured at each wave with the 9-item Center for Epidemiologic Studies Depression Scale (CES-D-9), a short form widely used in large-scale aging surveys (40). The score was computed by reverse-coding three positively worded items and summing all nine items (range 0–18; higher scores indicate more severe symptoms); internal consistency was satisfactory (Cronbach alpha = 0.73). Because the analysis is longitudinal, a two-factor structure-a negative-affect factor and a reverse-scored positive-affect factor-was specified and tested for longitudinal measurement invariance between the 2020 and 2023 waves before modeling change over time (Supplementary Table S1).

#### 2.3.2 Four indicators of the multilevel digital divide

Following the established multilevel framework (9, 10), four indicators were constructed at the 2020 baseline, each coded so that higher values indicate a deeper divide. First-level (access) divide: a binary indicator coded 1 if the respondent did not have home internet and 0 otherwise (41). Second-level (smartphone proficiency—the operational-skill facet) divide: the respondent's self-reported smartphone proficiency (original scale 1 = very unskilled to 5 = very skilled), reverse-coded so that higher values indicate lower proficiency; defined only among internet users, and conceptually distinct from problematic-use constructs that capture excessive engagement among connected users (42). Second-level (usage variety) divide: a count of online activities the respondent did not engage in, obtained by inverting an activity count, so that higher values indicate narrower use; defined only among internet users. Third-level (outcome) divide: a binary indicator of perceived adverse consequences of internet use across eight domains of daily life common to the 2020 and 2023 waves (21), coded 1 = one or more, 0 = none; this indexes the outcome level by adverse rather than realized beneficial outcomes of use. The two second-level indicators and the third-level indicator are defined only among internet users.

#### 2.3.3 Mediators: four indicators across three components of social connection

Four parallel mediators were operationalized within Holt-Lunstad's framework (24, 25): two structural indicators, one functional indicator, and one qualitative indicator, all measured contemporaneously with the exposure and again at follow-up. The first structural indicator, network monthly-contact count, summed 2 monthly-contact items (relatives and friends seen or heard from at least once a month; each 0–5; composite 0–10), indexing ego-centric network size and contact frequency. The second structural indicator, intergenerational contact frequency, was the reverse-coded mean across all living adult children of how often the respondent contacted each child in the past 12 months (range 1–5; higher = more frequent). The functional indicator, community-provided instrumental support, counted six community instrumental services-home visits, accompaniment to medical appointments, daily shopping assistance, in-home housework, senior meal provision, and day-care or senior day-stations (range 0–6; higher = broader availability); three further items (service hotline, legal aid, psychological counseling) were excluded as informational or emotional rather than instrumental support, since aggregating across sub-domains would obscure the instrumental pathway of interest; perceived availability is more consistently associated with depressive symptoms than received support (30–32). The qualitative indicator, intergenerational closeness, was the mean affectual closeness with children (1 = not close to 3 = close), averaged across all living adult children (43). The two structural indicators were entered separately rather than aggregated, given evidence that network breadth and dyadic intergenerational frequency relate to health via partially distinct pathways (37, 43). Estimating the four indicators jointly within a single framework provides a competitive test of which component of social connection, if any, mediates the prospective association of the digital divide.

#### 2.3.4 Covariates

Following prior literature (15, 33), all models adjusted for 2020 baseline covariates: gender, age and an indicator for age ≥ 75 years, educational attainment (three categories: illiterate; primary/junior high; high school or above), marital status, hukou status (rural vs. urban), number of co-residents, current employment, geographic region (Eastern, Central, Western), any of 23 chronic conditions, Activities of Daily Living and Instrumental Activities of Daily Living sum scores, and insurance enrolment.

### 2.4 Statistical analysis

Analyses began with descriptive statistics for all study variables across the three waves and unadjusted comparisons of 2023 depressive symptoms across baseline characteristics. Longitudinal measurement invariance of the CES-D-9 was then evaluated by comparing configural, metric, and scalar models across the 2020 and 2023 waves, with invariance judged by the criteria delta-CFI ≤ 0.01 and delta-RMSEA ≤ 0.015.

The primary analysis was a prospective lagged analysis. All models were estimated by ordinary least squares with heteroskedasticity-robust standard errors; the second- and third-level divides were analyzed among 2020 internet users, for whom the constructs are defined, and the access divide used the full sample. Prospective total effects were estimated by regressing 2023 CES-D-9 on each 2020 divide indicator, adjusting for baseline (2020) CES-D-9 and the covariate block; mediators were excluded from these models, as adjustment for the 2020 mediators would induce over-control. Mediation was assessed using the half-longitudinal approach of Cole and Maxwell (44), which recovers indirect effects from two lagged regressions while adjusting for the baseline values of both mediator and outcome. For each divide-by-mediator pair, the a-path regressed the 2023 mediator on the 2020 divide indicator, adjusting for the 2020 mediator and covariates; the b-path regressed 2023 CES-D-9 on the 2020 mediator, adjusting for the 2020 divide indicator, baseline CES-D-9, and covariates. Indirect effects (a × b) were tested with nonparametric bootstrap confidence intervals (5,000 resamples), reporting both bias-corrected and percentile 95% intervals as the primary basis for inference, following current mediation standards (45); the a- and b-paths were re-estimated within each resample to preserve their sampling covariance. As a stronger test of temporal ordering, we additionally estimated a prospective three-wave mediation in which usage variety (2018), instrumental support (2020), and depressive symptoms (2023) occupied successive waves, with all covariates measured at or before 2018; the a-path adjusted for the 2018 baseline of the mediator, and the b-path was estimated both controlling the outcome's exposure-wave baseline (2018) and, additionally, its mediator-wave baseline (2020).

As a follow-up analysis, moderation of the primary mediated pathway was examined for three prespecified moderators-hukou, living arrangement, and gender, each an established axis of health inequality among Chinese older adults along which both support resources and the mental-health returns to internet use are known to differ (39, 46, 47), by adding interaction terms on the a-path (usage x moderator) and the b-path (support x moderator); a significant interaction indicates that the corresponding path, and hence the indirect effect, varies across groups. To distinguish the resource-substitution and resource-multiplication accounts of any urban-rural difference, baseline instrumental support was compared across hukou groups. Robustness of the core mediation was assessed in the earlier window. Three further sensitivity analyses probed the robustness of the usage-variety result. To accommodate the complex survey design, the prospective models were re-estimated with CLASS post-stratification sampling weights and with standard errors clustered at the province (primary sampling unit) level. To gauge sensitivity to differential attrition across waves, they were re-estimated with inverse-probability weights derived from a baseline probit model predicting retention through 2023, after comparing baseline characteristics of retained and lost respondents. Finally, because several divide indicators and mediators were screened, we treated the analysis as involving multiple comparisons: the usage-variety pathway and its earlier-window replication were pre-specified as focal, and all screened estimates are reported in full so that readers can weigh them accordingly (Supplementary Table S2). The lagged design and adjustment for the baseline outcome mitigate, but cannot eliminate, residual confounding.

To separate between-person from within-person associations, and to address the concern that lagged regressions can conflate stable individual differences with within-person dynamics (48), we additionally estimated a random-intercept cross-lagged panel model (RI-CLPM) for the focal usage-variety–depression relationship across the three waves. Random intercepts absorbed time-invariant between-person differences while the autoregressive, cross-lagged, and contemporaneous parameters were estimated within persons; the model specification, fit, and key parameters are reported in Supplementary Tables S3, S3b; the focal within-person cross-lag was unchanged under both standardized and unstandardized parameterizations and whether the lagged paths were freely estimated or constrained equal across the unequally spaced waves. We further attempted to carry instrumental support within this random-intercept framework; because the three-wave RI-CLPMs involving instrumental support yielded inadmissible solutions, we used a fixed-effects dynamic-panel specification to isolate the within-person component of the mediated pathway (49, 50).

## 3 Results

### 3.1 Sample characteristics and measurement invariance

The conceptual model and the estimated pathway are summarized in Figure 2. The analytic sample for descriptive comparison comprised 5,914 older adults with complete 2020 baseline and 2023 outcome data. Half were women (50.1%), 12.2% were aged 75 or older, and 56.3% held rural hukou; 99.2% had ever been married and 67.8% lived with two or more co-residents (in households of three or more). Mean CES-D-9 scores were stable across waves (6.60, 6.66, and 6.56 in 2018, 2020, and 2023, respectively, on the 0–18 scale). In unadjusted comparisons, 2023 depressive symptoms were higher among women, those aged 75 or older, and rural residents (all *p* < 0.001; Table 1). Across waves, the proportion lacking home internet fell steadily (0.76, 0.49, and 0.41), while the smartphone-proficiency and usage-variety divides changed little among users (Table 2).

**Figure 2:**
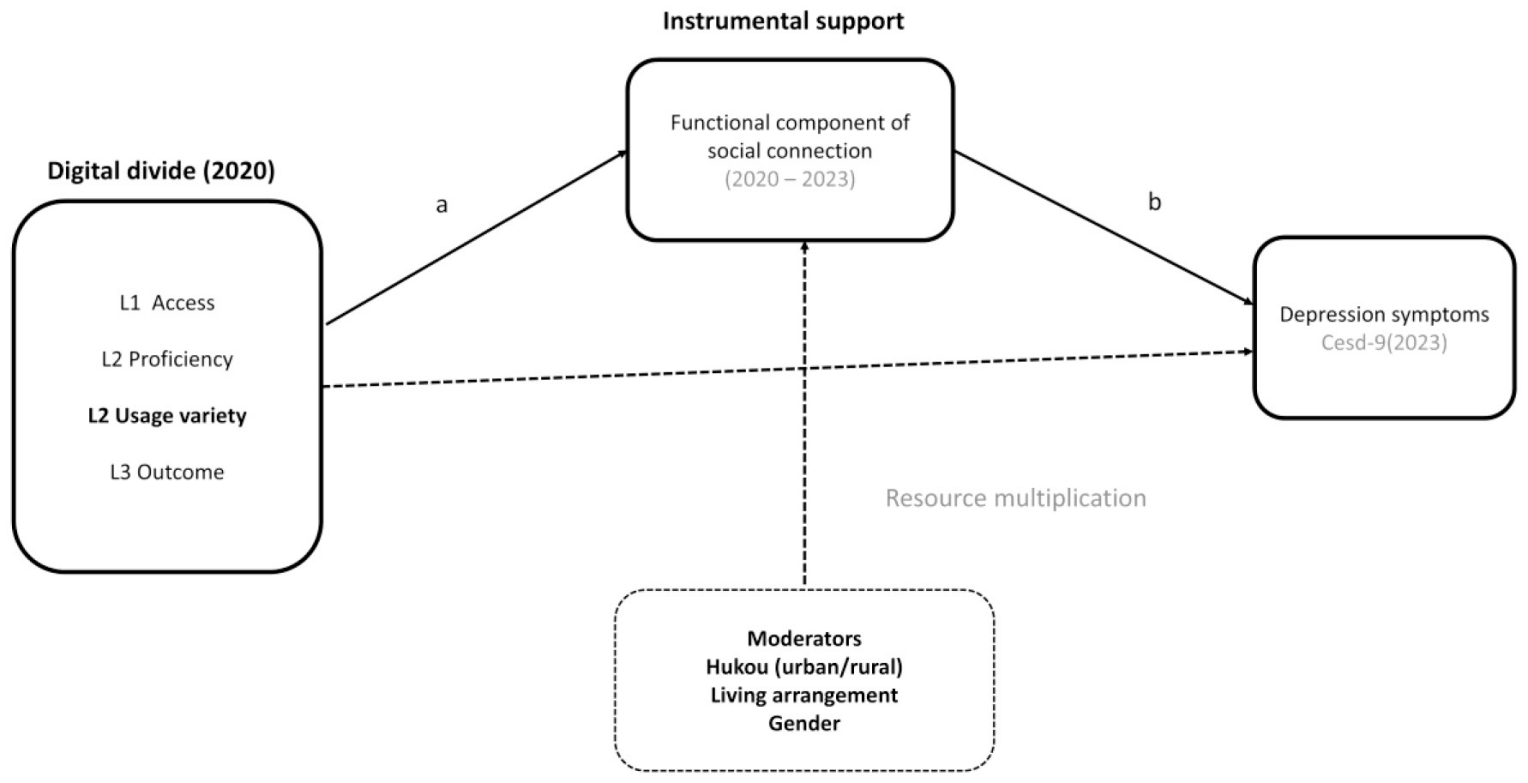
Conceptual model. Four indicators of the multilevel digital divide (2020) are modeled as prospective predictors of 2023 depressive symptoms. The functional component of social connection (instrumental support) is tested as the mediator (a- and b-paths) using a half-longitudinal specification; hukou, living arrangement, and gender are examined as moderators of the mediated pathway. The dashed line denotes the direct X → Y path (c′).

**Table 1:** Baseline (2020) sample characteristics and unadjusted 2023 CES-D-9 depressive symptoms (*N* = 5,914).

Characteristic	Level	*n* (%)	2023 CES-D-9, M (SD)	*p*
Gender	Female	2,965 (50.1)	6.71 (3.32)	<0.001
	Male	2,949 (49.9)	6.41 (3.29)	
Age ≥ 75 (2020)	<75	5,193 (87.8)	6.45 (3.34)	<0.001
	≥75	721 (12.2)	7.36 (2.91)	
Marital status	Not married	48 (0.8)	6.90 (4.05)	0.479
	Married	5,866 (99.2)	6.56 (3.30)	
Living arrangement	With others	4,010 (67.8)	6.49 (3.30)	0.020
	Alone	1,904 (32.2)	6.70 (3.31)	
Hukou	Urban	2,587 (43.7)	6.10 (3.26)	<0.001
	Rural	3,327 (56.3)	6.91 (3.29)	
Chronic condition	No	1,396 (23.6)	6.42 (3.25)	0.066
	Yes	4,518 (76.4)	6.60 (3.32)	

Percentages are column proportions within each characteristic. *p* values from *t*-tests (binary) or one-way ANOVA; the 2023 CES-D-9 is the outcome. All characteristics measured at the 2020 wave.

**Table 2:** Descriptive statistics of study variables across the 2018, 2020, and 2023 waves.

Variable	Wave	*n*	Mean (SD)
CES-D-9 (0–18)	2018	4,470	6.60 (3.10)
	2020	5,914	6.66 (3.27)
	2023	5,914	6.56 (3.30)
No home internet (0/1)	2018	4,668	0.76 (0.43)
	2020	5,914	0.49 (0.50)
	2023	5,914	0.41 (0.49)
proficiency divide (1–5)	2018	1,108	2.42 (0.85)
	2020	1,972	2.32 (0.86)
	2023	2,549	2.29 (0.86)
Usage variety divide (0–10)	2018	1,108	7.56 (1.84)
	2020	1,972	6.86 (2.17)
	2023	2,549	6.89 (2.06)

Higher values indicate a deeper divide. The third-level outcome divide was not measured in 2018. Second-level divides are defined only among internet users, hence their smaller *n*.

The two-factor CES-D-9 model fitted well in the configural specification (CFI = 0.986, RMSEA = 0.030, SRMR = 0.031). Imposing metric and then scalar constraints across the 2020 and 2023 waves produced negligible decrements in fit (delta-CFI = 0.001 and 0.003, respectively), satisfying conventional invariance criteria and confirming that the scale measured the same construct comparably over time (Supplementary Table S1).

### 3.2 Prospective total effects of the digital divide

After adjustment for baseline depression and covariates, the prospective total effects of the four divide indicators on 2023 depressive symptoms were largely null (Table 3). The first-level access divide (b = 0.005, 95% CI −0.131 to 0.141) and the second-level proficiency divide (b = 0.068, 95% CI −0.077 to 0.213) were not associated with subsequent symptoms, and the third-level outcome divide was likewise non-significant under the binary coding (b = −0.170, 95% CI −0.356 to 0.016). Because the higher-order divides are defined only among internet users, the access model necessarily spans users and non-users, whereas the proficiency, usage-variety, and outcome models are confined to users; the four coefficients therefore describe different populations and are not horizontally comparable. The access null accordingly bears only on whether baseline access predicts subsequent symptoms in the whole sample, not on the relative importance of the four levels, and the study's inference rests on the usage-variety effect estimated within the internet-user sample. The single robust exception was the second-level usage-variety divide, which predicted higher subsequent depressive symptoms (b = 0.069, 95% CI 0.008 to 0.131): older adults with a narrower range of online activity in 2020 had higher 2023 symptom scores, conditional on baseline symptoms. The baseline CES-D-9 was strongly predictive in every model (b approximately 0.59–0.62, *p* < 0.001), as expected for a lagged-outcome specification. The baseline CES-D-9 dominated each model, and the adjusted R^2^ ranged from 0.37 to 0.44; adding the divide indicator changed explained variance only negligibly (ΔR^2^ ≤ 0.002 in every model), underscoring the limited prospective contribution of the divide once baseline depression was held constant.

**Table 3:** Prospective total-effect associations between 2020 digital-divide indicators and 2023 CES-D-9 depressive symptoms: lagged OLS regression adjusting for the 2020 baseline outcome and confounders, with robust standard errors.

Variable	Model 1: Access	Model 2: Proficiency	Model 3: Usage	Model 4: Outcome
First-level access divide (2020)	0.005	—	—	—
	[−0.131, 0.141]			
Second-level smartphone proficiency divide (2020)	—	0.068	—	—
		[−0.077, 0.213]		
Second-level usage variety divide (2020)	—	—	0.069^*^	—
			[0.008, 0.131]	
Third-level outcome divide, any barrier (2020)	—	—	—	−0.170
				[−0.356, 0.016]
Baseline CES-D-9 (2020)	0.622^***^	0.588^***^	0.586^***^	0.607^***^
	[0.596, 0.648]	[0.542, 0.634]	[0.541, 0.632]	[0.572, 0.643]
Age (years, 2020)	0.002	−0.071^**^	−0.077^***^	−0.029
	[−0.019, 0.024]	[−0.115, −0.027]	[−0.122, −0.033]	[−0.060, 0.001]
Region fixed effects	Yes	Yes	Yes	Yes
Observations	5,914	1,828	1,828	1,828
Adjusted R^2^	0.436	0.370	0.371	0.419
ΔR^2^ (divide indicator)	0.000	0.000	0.002	0.001
Sample	Full	Users	Users	Users

Unstandardized coefficients with 95% confidence intervals in brackets. Each column is a separate lagged OLS regression reporting the prospective total effect of one divide indicator (mediators excluded), estimated with heteroskedasticity-robust standard errors. All models adjust for the baseline (2020) CES-D-9 and the full 2020 covariate block and include region fixed effects; age is shown as an exemplar covariate. Adjusted R^2^ is reported for each model, and ΔR^2^ is the change in explained variance attributable to adding the divide indicator. The access model uses the full analytic sample; the proficiency, usage, and outcome models are restricted to 2020 internet users. ^*^ *p* < 0.05; ^**^ *p* < 0.01; ^***^ *p* < 0.001.

### 3.3 Instrumental support mediates the usage-variety divide

Half-longitudinal mediation tested the four social-connection indicators in parallel for each divide indicator (Table 4). For the usage-variety divide, only the functional component mediated the association: a narrower range of use predicted lower subsequent instrumental support (a = −0.097), which in turn predicted higher depressive symptoms (b = −0.193), yielding a positive indirect effect (a × b = 0.019; bias-corrected bootstrap 95% CI 0.010 to 0.033). None of the structural or qualitative indicators mediated the usage divide (all bias-corrected bootstrap 95% confidence intervals included zero). Interpretation centers on the usage-variety divide—prespecified as focal and the only divide with a significant total effect (Table 3)—for which a mediation account is substantively meaningful. Testing all four components against every divide (Table 4), two additional indirect effects crossed the bias-corrected bootstrap threshold: access through instrumental support (0.016; 95% CI 0.005 to 0.031) and the outcome divide through contact frequency (0.011; 95% CI 0.002 to 0.028). Because neither divide showed a total effect—and because the outcome-divide path was not corroborated by the Sobel test (*p* = 0.057)—neither is interpreted as a substantive pathway. The proficiency divide showed no significant indirect effect through any component. The one prospective divide–depression association—that of the usage-variety divide—was thus statistically mediated specifically by lower practical, instrumental support, rather than by the frequency of contact or the closeness of relationships. In a prospective three-wave specification that ordered all three constructs across successive waves—usage variety (2018), instrumental support (2020), and depressive symptoms (2023) —with covariates measured at or before the 2018 exposure, the mediated pathway was reproduced. Controlling the outcome's exposure-wave baseline, the indirect effect was 0.040 (bias-corrected bootstrap 95% CI 0.022 to 0.066; *N* = 1,089); additionally controlling the outcome's mediator-wave baseline (2020) left it essentially unchanged at 0.031 (95% CI 0.016 to 0.055; *N* = 1,077), so the effect held whether or not the contemporaneous outcome was conditioned out (Supplementary Table S6). Because the third-level outcome divide was measured only from 2020, this prospective three-wave test was limited to the focal usage-variety pathway. Its agreement with the primary half-longitudinal estimate (0.019) and the 2018–2020 replication (0.014), despite a pre-pandemic exposure and a longer interval, indicates that the mediated association is robust to temporal specification.

**Table 4:** Half-longitudinal mediation of digital-divide effects on 2023 CES-D-9 through four components of social connection, with bootstrap confidence intervals.

Divide (X)	Mediator (M)	a	b	a × b	Bootstrap 95% CI [BC]
Access (L1)	Network contact (S)	−0.113	−0.012	0.0013	[−0.002, 0.006]
	Intergen. frequency (S)	0.013	−0.162	−0.0022	[−0.008, 0.002]
	Instrumental support (F)	−0.092	−0.170	0.0156^*^	[0.005, 0.031]
	Intergen. closeness (Q)	−0.015	−0.083	0.0013	[−0.001, 0.006]
Proficiency (L2)	Network contact (S)	−0.069	0.010	−0.0007	[−0.010, 0.004]
	Intergen. frequency (S)	0.005	−0.110	−0.0006	[−0.008, 0.002]
	Instrumental support (F)	−0.009	−0.203	0.0018	[−0.016, 0.020]
	Intergen. closeness (Q)	−0.013	0.189	−0.0024	[−0.015, 0.002]
Usage variety (L2)	Network contact (S)	−0.028	0.003	−0.0001	[−0.004, 0.002]
	Intergen. frequency (S)	0.036	−0.120	−0.0044	[−0.012, 0.003]
	**Instrumental support (F)**	**−0.097**	**−0.193**	**0.0186** ^*^	**[0.010, 0.033]**
	Intergen. closeness (Q)	0.002	0.170	0.0004	[−0.001, 0.005]
Outcome (L3)	Network contact (S)	−0.114	−0.007	0.0008	[−0.005, 0.008]
	Intergen. frequency (S)	−0.063	−0.178	0.0112^*^	[0.002, 0.028]
	Instrumental support (F)	0.066	−0.220	−0.0145	[−0.042, 0.007]
	Intergen. closeness (Q)	−0.020	−0.021	0.0004	[−0.007, 0.009]

Half-longitudinal mediation [Cole & Maxwell, (44)]. S, F, and Q denote structural, functional, and qualitative components of social connection (Holt-Lunstad). The a-path regresses the 2023 mediator on the 2020 divide indicator, adjusting for the 2020 baseline mediator and covariates; the b-path regresses 2023 CES-D-9 on the 2020 mediator, adjusting for the 2020 divide indicator, the 2020 baseline CES-D-9, and covariates; indirect = a × b. Bootstrap 95% CI [BC] is the bias-corrected interval from 5,000 resamples in which a and b are re-estimated jointly to preserve their covariance; percentile intervals (not shown) were materially identical. An asterisk marks an indirect effect whose bias-corrected 95% CI excludes zero. Covariates (all 2020): gender, age, education, marital status, living arrangement, hukou, ADL, IADL, chronic condition, employment, medical insurance, age ≥ 75, and region. The access divide uses the full analytic sample; the second- and third-level divides use the internet-user sample. Bold values denote statistically significant indirect effects (a × b) — those whose bias-corrected bootstrap 95% CI excludes zero.

### 3.4 Moderation, resource multiplication, and robustness across observation windows

The mediated pathway was not uniform across the cohort (Table 5, Panel A). It differed markedly by hukou: the indirect effect was present among urban older adults (a × b = 0.031) but essentially absent among rural older adults (a × b = −0.001), with a significant interaction on the a-path (0.112, *p* = 0.002) and a borderline interaction on the b-path (0.199, *p* = 0.051). Living arrangement moderated the b-path (interaction −0.225, *p* = 0.005), the indirect effect being larger among co-resident (0.029) than empty-nest (0.009) older adults, and gender showed a significant a-path interaction (−0.080, *p* = 0.025) but no b-path interaction and a weaker overall contrast. This urban-rural patterning was consistent with resource multiplication rather than resource substitution (Table 5, Panel B): at baseline, urban older adults reported substantially more instrumental support than their rural counterparts (difference 0.82, t = 9.39, *p* < 0.001), and the chain linking digital use to support and then to mental health was weaker in rural areas at both stages. Rather than compensating for scarce resources, greater usage variety was associated with a larger support advantage among those who already possessed more.

**Table 5:** Moderated mediation of the usage -> instrumental support -> depression pathway, and resource-multiplication evidence.

Moderator/level	a	b	a × b	Path interaction (*p*)
Panel A. Conditional indirect effects				
Hukou: urban	−0.121	−0.258	0.0311	a: 0.112^**^ (0.002)
Hukou: rural	−0.027	0.031	−0.0008	b: 0.199 (0.051)
Living: empty-nest	−0.093	−0.098	0.0091	a: −0.022 (0.562)
Living: co-resident	−0.114	−0.255	0.0290	b: −0.225^**^ (0.005)
Gender: female	−0.054	−0.170	0.0091	a: −0.080^*^ (0.025)
Gender: male	−0.131	−0.221	0.0290	b: −0.063 (0.407)
Panel B. Resource multiplication				
Baseline support, urban – rural	0.820			t = 9.39, *p* < 0.001
a-path interaction (usage x rural)	0.112			*p* = 0.002
b-path interaction (support x rural)	0.199			*p* = 0.051

Panel A reports group-specific a, b, and indirect effects; moderation is tested by path-specific interactions. Panel B: urban older adults begin with more instrumental support, and the chain is weaker in rural areas on both paths, consistent with resource multiplication. ^*^ *p* < 0.05; ^**^ *p* < 0.01.

This mediated pathway was not specific to a single interval. Estimated separately in the 2018-to-2020 replication window, the usage-to-instrumental-support-to-depression chain reproduced in sign and significance (a = −0.079, b = −0.176, indirect effect 0.014; bias-corrected bootstrap 95% CI 0.005 to 0.028, excluding zero; *N* = 1,523). The replication sample (*N* = 1,523) is larger than the corresponding 2018 internet-user cell in Table 2 (*N* = 1,108) because it requires complete data only across the 2018-to-2020 interval rather than complete 2023 follow-up, and its convergence with the main 2020-to-2023 result indicates that the functional mediation is robust across observation windows.

### 3.5 Between- vs. within-person associations

To assess whether the prospective associations reflect directional relationships rather than stable between-person differences, we re-estimated the usage-variety–depression relationship with a random-intercept cross-lagged panel model, which fit well (scaled χ^2^(5) = 8.13, p = 0.149; robust CFI = 0.998; robust RMSEA = 0.030; SRMR = 0.020) and in which intraclass correlations confirmed that a substantial share of variance was stable and between-person (usage variety 39%, depressive symptoms 34%). The between-person association was reproduced, as evidenced by a positive correlation between the random intercepts of usage variety and depressive symptoms (r = 0.56, *p* < 0.001). Accordingly, older adults with persistently narrower use had persistently higher depressive symptoms. At the within-person level the two constructs co-varied contemporaneously (within-wave regression 0.30, *p* = 0.004; disturbance correlation 0.28, *p* < 0.001), but the within-person cross-lagged (prospective) path was small and non-significant (standardized −0.01, 95% CI −0.11 to 0.10, *p* = 0.87) and remained so under both standardized and unstandardized parameterizations and whether the lagged paths were freely estimated or constrained equal across the unequally spaced waves (Supplementary Tables S3, S3b). The disappearance of a within-person prospective path once random intercepts are added is a characteristic result when cross-lagged models are re-estimated in this way (48), rather than a peculiarity of this cohort; the usage-variety association is therefore most consistent with a prospective between-person difference with a within-person contemporaneous counterpart. We also sought to incorporate instrumental support into the random-intercept framework. However, the trivariate and usage–support RI-CLPMs yielded inadmissible Heywood-type solutions, so the stable between-person component of instrumental support could not be reliably separated in these data (Supplementary Table S4). We therefore isolated the within-person component of the mediated pathway with a fixed-effects dynamic-panel specification, which removes all time-invariant between-person differences without requiring the mediator's trait to be identified (Supplementary Table S5). The within-person mediated effect was not detectable either contemporaneously or prospectively; the prospective estimate was imprecise (indirect effect = 0.012, 95% CI −0.029 to 0.123), so a within-person contribution cannot be excluded. Taken together with the significant aggregate mediation, this pattern is more consistent with a predominantly between-person association than with a detectable within-person prospective mediated association.

## 4 Discussion

### 4.1 Principal findings

Ordering the digital divide before depressive symptoms and conditioning on the 2020 baseline produced a markedly more restrained picture than the prevailing cross-sectional literature would predict. Of the four divide indicators examined, only the second-level usage-variety divide, that is, a narrower range of the activities older adults actually performed online, remained prospectively associated with depressive symptoms through 2023, primarily as a stable between-person difference; the first-level access divide, the second-level proficiency divide, and the third-level outcome divide showed no robust prospective association once baseline depression was held constant. The contrast with prior work is the central point. Cross-sectional studies of older Chinese adults have repeatedly linked the access and capability divides to more depressive symptoms (12, 13, 17, 18), and quasi-experimental evidence on access expansion has reported protective effects (14); in our lagged analysis, the access association fell to essentially zero, while the usage-variety facet, comparatively underweighted in the access-centered literature, emerged as the one prospectively associated with later symptoms.

This reordering has substantive implications for how the divide is conceptualized in later life. If the prospectively informative facet is the range of online activity rather than the binary fact of access or self-rated skill, then the mental-health stakes of the digital divide lie less in whether older adults are connected than in how fully they use what they are connected to. Read in this light, our results do not contradict the cross-sectional and quasi-experimental literature so much as locate its prospective core more precisely: the one prior strand that has emphasized usage breadth and variety (19, 20) is also the strand most consistent with what survives a lagged specification. We therefore treat the usage-variety finding not as proof that narrow use causes depression, but as the most defensible prospective signal in a multilevel framework that was constructed, by design, to give no facet priority. The remainder of the discussion asks how this signal is mediated and for whom it holds.

### 4.2 A differentiated, functional pathway

The mediating pathway behind this single durable association was specific. Among four indicators spanning the structural, functional, and qualitative components of social connection, only the functional component-instrumental support-mediated the usage divide, and it did so robustly, replicating in an earlier window. Neither the frequency of contact nor the closeness of intergenerational ties mediated the association. A narrow range of internet use was thus associated with higher later depressive symptoms in relation to lower availability of practical, day-to-day help, rather than to weaker emotional bonds or less frequent contact. This interpretation is consistent with the contemporary aging-services context, in which community instrumental services-home visits, accompanied medical appointments, shopping assistance, meal provision, and day-care-increasingly rely on online registration and messaging platforms for awareness and access (51); older adults with narrower use are less able to navigate to these resources. That the structural and qualitative components did not mediate the usage-variety association, despite being available to the same competitive test, strengthens the functional interpretation and points to instrumental support as the resource that accounted for the mediated association (30, 31).

The random-intercept model clarifies the level at which this association is located: it is carried mainly by stable between-person differences (r = 0.56) together with a within-person contemporaneous co-movement, with no detectable within-person prospective effect (Section 3.5). The usage-variety association is therefore best read as a robust prospective between-person difference with a within-person contemporaneous counterpart, rather than as evidence that within-person change in use precedes change in mood. This is a stronger inference than the cross-sectional associations that dominate this literature: exposure precedes the outcome, baseline depression is held constant, and the association replicates in an earlier window and in a prospective three-wave specification; what it does not license is a claim about within-person dynamics, which we do not make.

### 4.3 Resource multiplication

Why the functional pathway should be strongest where instrumental support is already abundant, rather than where it is scarce, is clarified by two competing accounts from the sociology of health inequality. On a resource-substitution logic, a resource matters most when other resources are lacking, so greater usage variety should help rural older adults-who have less instrumental support-the most; on a resource-multiplication logic, complementary resources compound, so it should most benefit those who already command the support infrastructure it draws on (38, 52). The moderation pattern adjudicates between them. The mediated chain from usage variety through instrumental support to depression was concentrated among urban and co-resident older adults and was essentially absent in rural areas-the reverse of what substitution predicts. The same pattern is visible at each link of the chain: urban older adults began with substantially more baseline instrumental support than their rural counterparts (a difference of 0.82 on the six-service count, *p* < 0.001), and the chain was weaker in rural areas at both stages-the a-path linking usage variety to support and the b-path linking support to symptoms. Where the underlying community-service infrastructure was thin, in other words, broader online activity was associated with little additional instrumental support, and hence with a correspondingly weaker association with depressive symptoms. On this evidence, digital engagement is associated with greater resources among the already-resourced rather than compensating for what disadvantaged older adults lack-a pattern with direct equity implications, since the groups least able to convert greater usage variety into instrumental support are precisely those with the fewest alternatives. The gender contrast was smaller and confined to a single link: gender moderated the a-path—the tie from usage variety to instrumental support—but not the b-path, and the overall indirect-effect contrast was weak. This is consistent with documented gender differences in how older Chinese adults mobilize instrumental support (39), though it does not by itself adjudicate substitution vs. multiplication; we therefore rest that adjudication on the hukou and living-arrangement contrasts and read the gender difference as a narrower, single-link qualification.

### 4.4 Policy implications

Our findings point to a specific policy logic. If the observed association between later-life digital inclusion and mental health is partly linked to instrumental support, and if this pattern is stronger where community-service infrastructure is more developed, then expanding connectivity or teaching basic skills may not, on their own, deliver equivalent benefits across groups. Two implications follow. First, because the facet prospectively associated with symptoms is the range of use rather than mere access, digital-inclusion programmes for older adults should move beyond getting people online toward supporting a fuller repertoire of activity-and should keep that activity anchored to tangible services, by maintaining parallel non-digital enrolment, notification, and booking channels for community instrumental services and by embedding assisted digital navigation in neighborhood- and village-committee infrastructure, so that digital exclusion does not cascade into service exclusion (41). Second, because the conversion of greater usage variety into support was concentrated where support was already plentiful, a uniform rollout could benefit the already-resourced more than the resource-poor, advancing SDG 3 (Good Health and Wellbeing) for some while undermining SDG 10 (Reduced Inequalities) for others. In rural settings in particular, where baseline instrumental support is low, digital-inclusion initiatives may need to be paired with investment in the human and service infrastructure that lets online engagement be translated into practical help; absent that investment, such initiatives could widen the very gap they are meant to close. Aligning later-life digital policy with both goals therefore means explicitly prioritizing the populations-here, rural and empty-nest older adults-for whom greater usage variety does not yet convert into instrumental support.

### 4.5 Limitations

Several limitations qualify these conclusions. First, the higher-order divides are defined only among internet users, and the longitudinal requirement of complete data across waves reduced the analytic samples, limiting power and the generalizability of those estimates. Second, like most population-survey research on digital engagement and mental health, all divide indicators, the mediators, and the outcome were self-reported, raising the possibility of common-method bias. The design incorporates two procedural safeguards recommended for this problem: the predictors and mediators (2020) and the outcome (2023) are separated by a three-year interval, so the prospective and mediated estimates cannot arise from same-occasion response consistency, and the exposure, mediator, and outcome use heterogeneous formats—inverted activity and service counts, a proficiency rating, and a symptom scale—that do not share a common scale artifact. These features mitigate but do not remove the concern, and self-reported use may still diverge from objective behavior; replication with device-level or administrative indicators would provide a more stringent test. Because the analytic sample was restricted to respondents with complete data across waves, non-random attrition could bias these estimates if loss to follow-up was related to both the digital divide and depression; the usage-variety estimate was therefore re-examined under inverse-probability-of-attrition weighting, under which it was essentially unchanged (b = 0.070), and under survey-weighted, province-clustered estimation, under which its magnitude was preserved (b = 0.074) although the confidence interval widened under conservative clustered inference, leaving significance borderline; its replication in the earlier 2018–2020 window further supports robustness (Supplementary Table S2).

Third, although the lagged design and adjustment for baseline depression reduce confounding relative to cross-sectional work, residual and time-varying confounding cannot be excluded, and the estimates should be read as strengthened associations rather than definitive causal effects. Fourth, the observation window (2018–2023) falls within the COVID-19 pandemic and its aftermath, when older adults' online activity, the availability of community instrumental support, and depressive symptoms were all perturbed; although the focal association also held with a pre-pandemic 2018 exposure, every outcome was measured during the pandemic era, so period effects cannot be separated from routine aging in these data. The pathway therefore requires confirmation in a fully post-pandemic cohort.

Relatedly, the lagged regressions, including the a- and b-paths of the mediation, blend between-person and within-person variation; re-estimating the focal usage–depression relationship with a random-intercept cross-lagged panel model located it primarily at the between-person level and, within persons, in contemporaneous rather than prospective co-variation (Section 3.5). The three-wave complete-case sample underlying this model (*N* = 877) was younger and more urban than respondents observed less completely (mean age 67 vs. 72; a markedly lower rural-hukou share), so the between-person association is estimated within a relatively advantaged subgroup and its generalizability to more disadvantaged older adults is uncertain. The within-person prospective path was not detectable; the study's minimum detectable effect was approximately standardized β = 0.12 at 80% power. Accordingly, the null estimate should not be interpreted as evidence that small within-person prospective associations are absent (Supplementary Table S3b). Taken together, the findings are more consistent with a predominantly between-person mediated association than with a detectable within-person prospective mediated association; however, the imprecision of the within-person estimate means that a within-person contribution cannot be excluded, given the study's half-longitudinal design (Supplementary Tables S4, S5). Similarly, the moderated pathways (Table 5) reflect between-person contrasts rather than within-person mediation dynamics, which were not the aim of this study. Fifth, the usage effect, while robust across specifications, is modest in magnitude. Sixth, the analysis concerns a single national cohort of older Chinese adults, and the resource-multiplication pattern in particular may reflect the specific urban-rural structure of support in China; replication elsewhere would clarify its generality.

## 5 Conclusion

Under a prospective design that conditions on baseline depression, the apparent mental-health harms of the digital divide among older adults largely attenuate, with one durable exception: a narrow range of internet usage is prospectively associated with higher depressive symptoms; this association was statistically mediated by lower instrumental social support. This functional pathway was stronger in resource-rich subgroups-urban and co-resident older adults-consistent with resource multiplication rather than substitution. Converting later-life digital inclusion into mental-health gains is therefore likely to require strengthening the underlying support infrastructure, especially in rural areas, rather than expanding access or skills in isolation-an equity-centered approach that serves both SDG 3 and SDG 10 in step with the WHO Decade of Healthy Aging.

## Data Availability

Publicly available datasets were analyzed in this study. This data can be found here: http://class.ruc.edu.cn.
