# Supplementary material for: Beyond access: usage variety, instrumental support, and later-life depression in a prospective study of Chinese older adults
Source: Front Public Health. 2026 Sep 30;14:1895216. doi: 10.3389/fpubh.2026.1895216 (PMC13662110; doi:10.3389/fpubh.2026.1895216)
Supplement: Supplementary file 1 [file Table_1.docx]

**Supplementary Material**

*Beyond Access: Usage Variety, Instrumental Support, and Later-Life Depression in a Prospective Study of Chinese Older Adults*

**Supplementary Table S1.** Two-wave longitudinal measurement invariance of the 9-item CES-D depressive symptoms scale (correlated two-factor model) across the 2020 and 2023 waves of CLASS (N = 5,914).

| **Model** | **χ²** | **df** | **CFI** | **RMSEA** | **SRMR** | **ΔCFI** | **ΔRMSEA** | **Decision** |
| --- | --- | --- | --- | --- | --- | --- | --- | --- |
| Configural | 775.2 | 120 | 0.986 | 0.030 | 0.031 | — | — | **Reference** |
| Metric | 808.2 | 125 | 0.985 | 0.030 | 0.031 | 0.001 | 0.000 | **Supported** |
| Scalar | 962.9 | 134 | 0.982 | 0.032 | 0.031 | 0.003 | 0.002 | **Supported** |

*Note. Longitudinal measurement invariance across the 2020 and 2023 waves. Metric and scalar constraints produced negligible decrements in fit (ΔCFI ≤ 0.003, ΔRMSEA ≤ 0.002), judged against the criteria ΔCFI ≤ 0.01 and ΔRMSEA ≤ 0.015; scalar invariance is supported.*

**Supplementary Table S2.** Sensitivity analyses for the prospective digital-divide → 2023 depression associations.

**Panel A.** Design-based replication of Table 3 with CLASS post-stratification survey weights (released with the 2023 wave; urban–rural × sex × age) and standard errors clustered at the province (primary sampling unit) level.

| **Digital-divide indicator (2020)** | **b** | **95% CI** |
| --- | --- | --- |
| First-level access divide | −0.038 | [−0.291, 0.215] |
| Second-level proficiency divide | 0.033 | [−0.183, 0.249] |
| Second-level usage-variety divide | 0.074 | [−0.026, 0.174] |
| Third-level outcome divide | −0.104 | [−0.506, 0.298] |
| *Observations (access / proficiency / usage / outcome)* |  | 5,914 / 1,828 / 1,828 / 1,828 |

*Note. Entries are coefficients with 95% confidence intervals in brackets; each indicator is estimated in its own lagged model adjusting for baseline (2020) CES-D-9 and the full covariate block. Specification: weighted + province-clustered (27–28 clusters). The usage-variety estimate (b = 0.074) closely matches the main analysis (b = 0.069); the wider interval reflects conservative province-clustered inference.*

**Panel B.** Attrition sensitivity for the usage-variety total effect: complete-case versus inverse-probability-of-attrition-weighted (IPW) estimates.

| **Specification** | **Usage-variety b** | **95% CI** | **N** |
| --- | --- | --- | --- |
| Complete case (robust) | 0.069* | [0.008, 0.131] | 1,828 |
| IPW for attrition | 0.070* | [0.008, 0.131] | 1,828 |

*Note. Baseline universe = 2,519 internet users (1,828 retained through 2023; 691 lost). IPW are stabilised inverse probabilities from a probit of retention on the baseline exposure, baseline CES-D-9, and the full covariate block, trimmed at the 1st/99th percentiles. Although lost respondents had modestly lower baseline usage variety and were older, the usage-variety estimate is essentially unchanged after IPW (0.069 → 0.070), indicating the result is not driven by differential attrition. * p < 0.05.*

**Supplementary Table S3.** Random-intercept cross-lagged panel model (RI-CLPM) of usage variety and depressive symptoms across the 2018, 2020, and 2023 waves of CLASS. Random intercepts absorb stable between-person differences; the within-person components carry the autoregressive, cross-lagged, and contemporaneous parameters.

| **Parameter** | **Estimate** | **SE** | **p** | **95% CI** |
| --- | --- | --- | --- | --- |
| ***Model fit*** | | | | |
| Scaled χ² (df = 5) | 8.13 | — | .149 | — |
| Robust CFI | 0.998 | — | — | — |
| Robust RMSEA | 0.030 | — | — | — |
| SRMR | 0.020 | — | — | — |
| ***Between-person (random intercepts)*** | | | | |
| Correlation of random intercepts (usage, depression) | 0.558 | 0.141 | < .001 | [0.282, 0.833] |
| ICC, usage variety | 0.392 | — | — | [0.201, 0.582] |
| ICC, depressive symptoms | 0.336 | — | — | [0.195, 0.477] |
| ***Within-person*** | | | | |
| Contemporaneous within-wave regression | 0.30 | — | .004 | — |
| Disturbance correlation (contemporaneous) | 0.28 | — | < .001 | — |
| **Cross-lag: usage → subsequent depression (focal)** | **−0.01** | **—** | **.87** | **[−0.11, 0.10]** |

*Note. RI-CLPM estimated by robust maximum likelihood on three waves (2018, 2020, 2023). Random intercepts absorb stable between-person variance; intraclass correlations (ICCs) give the between-person share of variance (usage variety 39%, depressive symptoms 34%). The focal within-person cross-lagged path (usage → subsequent depression) is small and non-significant and remains so under both the standardised parameterisation reported here and the unstandardised parameterisation in Supplementary Table S3b. In the reported model the lagged (autoregressive and cross-lagged) paths were constrained equal across the two intervals, so a single coefficient was estimated for each lagged relationship. An equal-interval constraint equating the four lagged coefficients across the 2- and 3-year intervals was tested against the freely-estimated model by a scaled (Satorra–Bentler) difference test and not rejected (Δχ²(4) = 6.79, p = 0.147); the freely-estimated model itself fit well (robust χ²(1) = 1.19, p = 0.28). The focal conclusion therefore does not depend on the unequal wave spacing. Estimates are standardised coefficients unless otherwise noted; ICC = intraclass correlation; — = not applicable.*

**Supplementary Table S3b. Full parameter estimates for the random-intercept cross-lagged panel model (RI-CLPM) of usage variety and depressive symptoms (2018, 2020, 2023 waves of CLASS).**

| **Parameter** | **Estimate** | **SE** | **p** | **95% CI** |
| --- | --- | --- | --- | --- |
| **Within-person dynamics (unstandardised; lagged paths constrained equal across the 2018→2020 and 2020→2023 intervals)** | | | | |
| **Usage autoregression (usageₜ₋₁ → usageₜ)** | **0.613** | **0.051** | **<.001** | **[0.514, 0.712]** |
| **Depression autoregression (depₜ₋₁ → depₜ)** | **0.425** | **0.065** | **<.001** | **[0.298, 0.552]** |
| **Cross-lag: usage → subsequent depression (focal)** | **−0.014** | **0.081** | **.865** | **[−0.172, 0.145]** |
| **Cross-lag: depression → subsequent usage (reverse)** | **−0.003** | **0.022** | **.876** | **[−0.047, 0.040]** |
| **Within-person contemporaneous (disturbance) correlation, by wave** | | | | |
| **2018** | **0.130** | **0.086** | **.128** | **[−0.037, 0.298]** |
| **2020** | **0.010** | **0.057** | **.867** | **[−0.103, 0.122]** |
| **2023** | **0.281** | **0.045** | **<.001** | **[0.193, 0.369]** |
| **Between-person (random intercepts)** | | | | |
| **Usage-variety RI variance** | **1.080** | **0.269** | **<.001** | **[0.554, 1.606]** |
| **Depressive-symptom RI variance** | **3.562** | **0.786** | **<.001** | **[2.022, 5.101]** |
| **RI correlation (usage variety, depression)** | **0.558** | **0.141** | **<.001** | **[0.282, 0.833]** |
| **ICC, usage variety** | **0.392** | **0.097** | **<.001** | **[0.201, 0.582]** |
| **ICC, depressive symptoms** | **0.336** | **0.072** | **<.001** | **[0.195, 0.477]** |
| **Model-implied within-person same-wave regression (usage → depression), by wave** | | | | |
| **2018** | **0.267** | **0.179** | **.136** | **[−0.084, 0.619]** |
| **2020** | **0.041** | **0.109** | **.706** | **[−0.173, 0.256]** |
| **2023** | **0.297** | **0.102** | **.004** | **[0.096, 0.497]** |

*Note. RI-CLPM estimated by robust maximum likelihood (MLR) on the three-wave complete-case sample (N = 877). Lagged (autoregressive and cross-lagged) paths were constrained equal across the 2018→2020 and 2020→2023 intervals; the constraint was not rejected by a scaled (Satorra–Bentler) difference test against the freely-estimated model (Δχ²(4) = 6.79, p = 0.147). Model fit (robust): scaled χ²(5) = 8.13, p = .149; CFI = 0.998; RMSEA = 0.030; SRMR = 0.020. All variance components were positive (no Heywood cases). For the focal prospective cross-lag, the minimum detectable within-person effect at 80% power was approximately standardized β = 0.12. The analysis therefore had limited power to exclude smaller within-person prospective associations, and the null cross-lag should not be interpreted as evidence that a small effect is absent.*

**Supplementary Table S4.** Attempts to incorporate instrumental support into a random-intercept cross-lagged framework across the 2018, 2020, and 2023 waves of CLASS.

| **Model** | **Instrumental-support random-intercept variance (SE)** | **Between-person correlation(s) with support** | **Admissible** |
| --- | --- | --- | --- |
| Trivariate RI-CLPM (usage, support, depression) | 0.03 (0.91) | usage 3.17; depression 2.84 | No |
| Bivariate: usage variety ↔ support | −3.98 (46.7) | 1.27 | No |
| Bivariate: support ↔ depressive symptoms | 2.25 (0.20) | −0.35 | Yes |

*Note. Random-intercept cross-lagged panel models estimated by robust maximum likelihood on three waves (2018, 2020, 2023). In the three-variable model and when instrumental support was paired with usage variety, the instrumental-support random intercept numerically converged to improper (Heywood-type) solutions: its variance collapsed toward zero or turned negative, its standard errors were one to two orders of magnitude larger than the estimates (e.g., 0.03 [SE 0.91]; −3.98 [SE 46.7]), and between-person correlations involving support exceeded one. These diagnostics indicate that the stable support variance component could not be reliably separated from these data at three waves.The instability arose because, once its covariance with the highly stable usage-variety intercept was partialled, the residual stable (trait) variance in three-wave support was too small to identify, an empirical-underidentification pattern rather than a substantive zero. The component was admissible only when instrumental support was paired with depressive symptoms. Entries are unstandardised random-intercept variances (SE) and standardised between-person correlations; |r| > 1 is inadmissible.*

**Supplementary Table S5.** Within-person mediated effect of the usage-variety pathway (usage variety → instrumental support → depressive symptoms): fixed-effects dynamic-panel estimates.

| **Specification** | **a** | **b** | **Indirect a×b (95% CI)** | **First-stage F** | **N** |
| --- | --- | --- | --- | --- | --- |
| First differences (contemporaneous) | +0.033 | −0.244 | −0.008 (−0.024, 0.006) | — | 2,583 |
| Anderson–Hsiao IV (prospective) | −0.081 | −0.151 | +0.012 (−0.029, 0.123) | 29–120 | 846 |

*Note. Fixed-effects dynamic-panel estimates that remove all time-invariant between-person differences by first-differencing and do not require the mediator’s stable component to be identified (Allison, Williams & Moral-Benito, 2017). The prospective specification uses the Anderson–Hsiao estimator (Anderson & Hsiao, 1981): after first-differencing, each lagged differenced regressor (Δinstrumental support and ΔCES-D-9 at the 2020 wave) is instrumented by its 2018 level (the twice-lagged level). First-stage F statistics were 58.0 (a-path support), 119.8 (b-path depression) and 29.3 (b-path support); the minimum first-stage F of 29 indicates the instruments are not weak, so the wide interval reflects genuine uncertainty about the within-person effect rather than weak identification; because it nonetheless reaches 0.12, a within-person contribution cannot be excluded. Confidence intervals are bias-corrected person-cluster bootstrap (2,000 resamples). N = person-intervals (contemporaneous) or persons (prospective). Coefficient signs follow the divide coding (higher = narrower use).*

**Supplementary Table S6.** Prospective three-wave mediation (usage variety 2018 → instrumental support 2020 → depressive symptoms 2023).

| **Specification** | **a** | **b** | **c′** | **Indirect a×b (95% CI)** | **N** |
| --- | --- | --- | --- | --- | --- |
| Primary (all baselines ≤ 2018) | −0.115 | −0.348 | +0.063 | 0.040 (0.022, 0.066) | 1,089 |
| Sensitivity (b-path also adj. depression 2020) | −0.116 | −0.268 | +0.049 | 0.031 (0.016, 0.055) | 1,077 |

*Note. Sample: respondents for whom usage variety was defined at the 2018 exposure wave. Covariates (measured at or before 2018): gender, age, education, marital status, hukou, co-residence, employment, region, chronic conditions, ADL/IADL, insurance. Because the third-level outcome divide was measured only from 2020, the analysis was limited to the focal usage-variety pathway. The two rows differ only in the outcome baseline entered in the b-path: the first controls the exposure-wave baseline (2018); the second additionally controls the mediator-wave baseline (2020), which is post-exposure relative to the 2018 exposure and removes the contemporaneous support–depression covariation. The indirect effect excludes zero under both baseline choices, so it does not depend on whether the mediator-wave outcome is conditioned out. Confidence intervals are bias-corrected bootstrap (5,000 resamples). Coefficient signs follow the divide coding (higher = narrower use).*

**Supplementary References**

Allison PD, Williams R, Moral-Benito E. Maximum likelihood for cross-lagged panel models with fixed effects. Socius. 2017;3:1–17.

Anderson TW, Hsiao C. Estimation of dynamic models with error components. J Am Stat Assoc. 1981;76(375):598–606.
